# Key Influencing Factors and Optimization Strategy of Epidemic Resilience in Urban Communities—A Case Study of Nanjing, China

**DOI:** 10.3390/ijerph19169993

**Published:** 2022-08-13

**Authors:** Peng Cui, Yi Liu, Xuan Ju, Tiantian Gu

**Affiliations:** 1 Department of Engineering Management, School of Civil Engineering, Nanjing Forestry University, Nanjing 210037, China; 2 Department of Engineering Management, School of Mechanics and Civil Engineering, China University of Mining and Technology, Xuzhou 221116, China

**Keywords:** COVID-19, urban community, epidemic resilience, ISM, PROMETHEE II

## Abstract

COVID-19 has posed a significantly severe impact on both people’s lives and the global economic development. Increasing the community epidemic resilience will considerably improve the national public health emergency response capacity from bottom to top. This study identifies the influencing factors of community epidemic resilience through systematic literature review under the 4R framework, then obtains the relationships of influencing factors through Interpretive structural model, and finally assesses the performance of epidemic resilience using PROMETHEE II method through empirical cases in Nanjing, China. The results show that: (1) Eight factors influencing the epidemic resilience of community are identified, and the economic level plays the root role; (2) Community epidemic resilience can be improved from robustness, rapidity, redundancy and resourcefulness aspects; (3) Through the empirical analysis, the epidemic resilience ranking of community can be displayed (Community D > T > S > F); (4) Additionally, the performance and sensitivity analysis of influencing factors in each community can be demonstrated. (5) Finally, four implications are proposed, namely, allocating public resources rationally, significantly increasing the economic level, ensuring the accuracy of information delivery and conducting disaster learning.

## 1. Introduction

The COVID-19 pandemic in the early 2020 has posed a serious threat to the whole world [1]. Alizadeh et al. defined the COVID-19 pandemic as the greatest challenge that humanity has ever faced since the Second World War [2]. Studies have found that the spread of novel coronavirus has greatly impacted numerous people from over 200 countries [3]. Simultaneously, this epidemic has also caused a series of social, economic, and environmental impacts worldwide [4]. In addition to the threat on physical conditions, the COVID-19 also triggers a wide range of psychological problems, such as panic [5]. As of 13 July 2022, there were estimated 555 million cumulative diagnoses and 6 million cumulative deaths worldwide, with 4 million cumulative diagnoses and 22 thousand cumulative deaths in China (Data source: CDC · WHO · ECDC · Wikipedia · New York Times · China Health Commission).

To respond to the COVID-19, most of the foreign governments have enforced border shutdowns, travel restrictions and quarantine [6]. Given the characteristic conditions and the existence of a democratic and centralized system in China, epidemic prevention and control measures are unique from other countries. In China, decisions made by the central and local governments are imposed on the public to restrict their mobility, for instance, the self-isolation policies, which have caused major socio-economic implications [7]. The Chinese government also enacts strict restrictions and policies to prevent people from gathering in groups [1]. The community, as the basic urban unit, is a common gathering place for people, which should raise more attention during the transmission of COVID-19. Increasing the acceptance of the vaccination program is also crucial [8]. The community system in China is based on political parties and residents’ committees, as grassroots mass self-governance organizations, are at the heart of community governance [9]. In the face of the COVID-19, China emphasizes the deterrent role of the community in prevention and control of the epidemic. The community is regarded as a protective net to effectively contain the spread of the virus. Since the outbreak of COVID-19, China has consistently implemented effective prevention and control measures. But unavoidably, sporadical cases still occur occasionally in some communities. For example, in December 2020, Yin in Shenyang, who was infected and supposed to conduct a seven-day quarantine, was yet left out in the community, closely contacting with approximately 12,000 people, with at least 27 infected. Another case is Mao, in July 2021, who left his neighborhood in Nanjing for Yangzhou without permission, resulting in the isolation and containment of more than 3000 people, with 47 confirmed cases among his close contacts. Thus, the outbreak management regarding COVID-19 should not be blindly optimistic, and sufficient attention should be paid at the community level.

Community resilience includes the prevention and response to natural disasters and major social emergencies, and the restoration and even leapfrogging of community functions after disasters [10,11]. A resilient community is characterized by robustness, redundancy, rapidity and resourcefulness and tends to suffer less while recover faster from the same shocks and stresses than a traditional one [11]. With the development of resilience theory, resilience has been widely applied in urban disaster prevention and reduction and emergency management. Adequate pre-epidemic social resilience plays a crucial role in outbreak response, post-disaster recovery and reconstruction. For example, trust in community managers from residents can significantly enhance the efficiency of outbreak prevention and control [12]. Generally, urban resilience includes three aspects [13]. Firstly, the ability of a city to maintain basic functions without extra external assistance when it is exposed to an emergency. Secondly, the ability of a city to recover in time. And thirdly, the ability of a city to gain experience from the previous disturbances and prepare for possible future disasters. Similarly, urban epidemic resilience is critical in the face of various disasters by identifying repetitive and procedural laws from a large number of uncertain factors, turning some uncertain work in emergencies into preventive management work in normal state, and focusing limited power on the most important aspects [1,14,15,16,17].

Identifying a systematic list of the influencing factors is the key to improving the resilience of urban community [18,19,20]. Accordingly, in existing literature, multiple dimensions of the urban community resilience have been identified. In fact, many researchers begin with cognition of the resilience in urban communities. For instance, knowledge in preparation for incidents, knowledge of strategic planning and awareness of the depth of the economic crisis are being considered [21,22,23]. All these influencing factors can be considered as perception. Given that is highly contagious, ignoring the importance of perception may imperil the public [24]. Faced with the outbreak of COVID-19, Galbusera et al. identified critical infrastructures as certainly important factors, such as essential needs, protective equipment, public health resources and emotional resources [12,21,22,25,26]. Furthermore, government is also one of the essential influencing factors of epidemic resilience in urban communities. Chu, Zhen et al. pointed out that urban governance capacity is an essential factor affecting the prevention and control of epidemic, such as government guidance, central government regarding the workforce, financial subsidies and material resources [22,27]. HEATH C et al. also considered the psychological health through spatial regression analysis, which includes well-being of nursing home staff and psychological attributes [28]. CHEN Y et al. argued that the free flow of information is an essential criterion fostering the resilience, thus adding a new dimension [26]. Despite of these major influencing factors, PENACOBA C et al. found that resilience during the COVID-19 pandemic has been negatively associated with pressure, anxiety, physical fatigue, or work performance while positively associated with happiness, high quality of life, or physical functionality [29]. However, few of these studies have presented a systematic list of the influencing factors about the urban community resilience during the outbreak of COVID-19, which is the key point in this paper.

The aforementioned studies have contributed greatly on the assessment of epidemic resilience in urban communities, but a few gaps still remain to be bridged: (1) More technical measures should be implemented than management measures; (2) There is a lack of systematic framework among influencing factors of community resilience; (3) Few research has been conducted on the source factors that affect community resilience under the epidemic situation as well as the relationship among these factors.

To fill these gaps, this study aims to discover the relationships between the factors affecting the community resilience through a systematic framework, which would be applied to the real community for experimental analysis to provide policy-makers with useful empirical evidence and reference regarding preparations for future incidents such as outbreaks of infectious diseases.

## 2. Materials and Methods

(1) This paper uses systematic literature review (SLR) for factor screening and combines principal component analysis (PCA) for factor selection by dimensionality reduction. The integrative review is the broadest type of research review method, which allows a comprehensive understanding about the phenomenon of concern [30]. Moreover, SLR can be applied to provide in-depth answers to specific questions from a multi-disciplinary perspective [30,31]. PCA is commonly used to reduce the dimensionality of data by introducing uncorrelated variables to separate the mostly correlated variables into further dimensions, with the principal component explaining the most variance [32,33]. It can effectively investigate the underlying relationship among the influencing factors [1,34]. Its main steps include original data standardization processing, correlation matrix drawing among indicators, solving eigenvalues and eigenvectors and contribution rate of each component and cumulative contribution rate calculation [16].
(2) There are several ways to investigate the source sexuality factor. The interpretative structural model (ISM) is simpler to implement than other methods, which allows the demonstration of the internal structure of a system by processing known but intricate system–element relationships [35]. Therefore, ISM is chosen to obtain the factor hierarchy in this paper.
(3) The PROMETHEE method uses a priority function to depict the priority of a finite number of alternatives in terms of attributes, and ranks the superiority of each alternative according to the gaps between the values of the attributes. Compared to other multicriteria decision making methods, the PROMETHEE method is simple and straightforward to implement and can thus be easily understood by decision makers. This makes it one of the predominantly used decision method for solving multi-attribute decision problems [14].
(4) Graphical Analysis for Interactive Aid (GAIA) is a descriptive helper method for PROMETHEE. It is a three-dimensional visual representation of the similarity between decision criteria and decision objectives. Each indicator is made axis in a space, and the projection length of the community on each axis indicates the advantage (or deficiency) degree of the indicator represented by the community on the axis [14].

**Figure 1:**
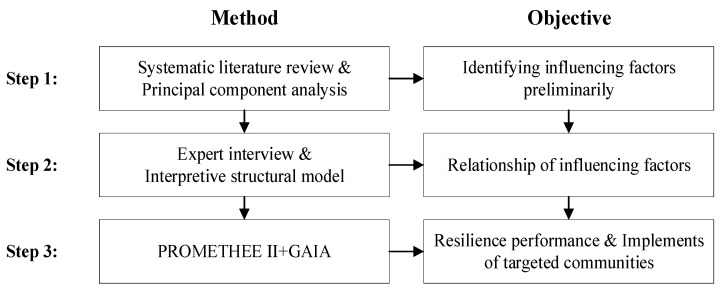
The organization of this study.

Based on the above methods, A combined theoretical and practical approach is proposed as shown in Figure 1. Firstly, the key influencing factors are screened through SLR. Secondly, a secondary screening and classification of the influencing factors are carried out using a questionnaire method with PCA. The obtained factors are then divided on the 4R dimensions. And after scoring the weights by the experts, a hierarchical model of influencing factors of urban resilience is constructed using the ISM. Finally, using PROMETHEE II along with GAIA, a case study is conducted in four communities in Nanjing. The paper will be organized into the following sections. 

### 2.1. Definitions of Epidemic Resilience and 4R Characteristics

Community resilience can be defined as: (1) the ability to maintain basic functions in the face of the uncertainty and perturbation of COVID-19; (2) the requirement for various resources, including human and material resources, as well as government policies to complement epidemic preparedness; (3) the governance in epidemic situations which emphasizes self-organization, self-adaptation and self-recovery, rather than reliance on external assistance.

4R characteristics of resilience include robustness, redundancy, rapidity and resourcefulness, each of which indicates an aspect of the preparedness and mitigation capacity that communities demonstrate when responding to disasters [36]. The more significant the above 4R characteristics are in a community, the more resilient the community is. Referring to Bruneau et al., the definitions in this paper are shown below.

**Robustness**: The ability to considerably maintain normal functioning and stable operating conditions after exposing to epidemic. Increasing the system’s robustness will significantly reduce the recovery time and thus prevent or minimize severe disruptions to the community [37]. 

**Rapidity**: The response of community to disasters, and the speed and efficiency of reconstruction and recovery from the epidemic. As an essential element of the epidemic resilience, it is closely related to the severity of the disaster, the availability and amount of the resources provided and the ability of the community to manage and operate the system [38]. 

**Redundancy**: The ability to maintain the original functional state through backup or other alternative systems, despite of the damaged systems of the community during the epidemic. The redundancy allows a community to maintain an acceptable level of service by providing additional systems [38]. 

**Resourcefulness**: The capacity that allocates resources and the ability to evaluate the performance of resilience of a community during the epidemic [39]. It also refers to the ability of the community to discover problems, thus take following actions immediately by mobilize resources. This is highly linked to human, social, and government management [40].

### 2.2. Identification of Influencing Factors

#### 2.2.1. Screening of Influencing Factors Based on SLR

“COVID-19” “community” “Resilience/resilient” are used as keywords to search in the two mainstream databases of ScienceDirect and Wiley Online Library, from which all retrieved English literature are screened and verified. In addition, all the studies are based on advanced search, limited to title, keywords and abstract. Only English language is accepted and the majority of the literature are journals. Moreover, the publication time is set from 2019 to 2022.

This paper formulated the following strategies for multiple screening of existing articles (Zhang et al., 2022) [41], including: (1) Repetitive screening. An article may belong to more than one database. For example, articles published by ScienceDirect may be indexed by OBCC, in which case the elimination process needs to be repeated to ensure that there are no duplicated articles collected; (2) Title screening. Title screening requires that titles are read to filter articles which are clearly not relevant to the assessment of community resilience of COVID-19; (3) Abstract screening. The abstracts were read to delete articles that are not relevant to the resilience of the community; (4) Full-text screening. The purpose is to read the full text of an article to increase accuracy; (5) Reference screening. This study can be supplemented by collecting missing articles from the references cited in the previous selected articles.

By using the above-mentioned index selection method, 45 articles related to evaluation indicators of resilience of urban communities during the outbreak of COVID-19 are finally determined in this paper. Based on the above research basis, word frequency statistics of the first and second level indicators in the above 45 articles are conducted. The indicators with occurrence frequency ≥ 3 are extracted and sorted out. The articles selected as well as the indicators are shown in Appendix A.

#### 2.2.2. Streamlined Process and Determination of Influencing Factors

The influencing factors listed in Appendix A are preliminarily screened based on the questionnaire shown in Appendix B. Firstly, all the indicators searched by SLR method are summarized and influential factors with frequency of less than 3 are eliminated. Secondly, the influencing factors obtained after screening are classified on the 4R framework. Then, the influencing factors with the same meaning are integrated and summarized into 21 questions. Finally, statistical analysis of the questionnaire is conducted. The first part of the questionnaire collects the basic statistically related information, such as gender and residency, while the second part asks questions corresponding to 21 influencing factors. The five-level Likert scale method is used to score the influencing factors of resilience, including specifically these following response options, completely non-conforming, not quite conforming, general, basically conforming, and completely conforming. 

Based on the 30 valid online questionnaires collected, PCA method is adopted to screen the influencing factors. With exclusion of the secondary influencing factors of which the frequency is lower than 3, a total of 21 influencing factors are obtained, which correspond to Q1~Q21 in the questionnaire respectively. The questionnaire results are summarized, and the Pearson correlation coefficient is used to calculate the correlation coefficient between the influencing factors as shown in Table 1.

It can be noted from Table 1 that the cumulative contribution rate of the first 8 principal components reaches 84.8%, which can fairly explain most of the differences in the 21 resilience factors in the original data. Therefore, eight factors are considered as the key influencing factors of epidemic resilience in urban communities as shown in Table 2.

The above eight influencing factors are classified by 4R characteristics as follows.

Economic level and social network are considered to belong to robustness. Firstly, economic level is closely related to people’s lives and thus can greatly influence the operation of the urban epidemic resilience [42]. Meanwhile, economic conditions directly influence the level of financial support provided by the government in key epidemic areas [27]. Social networks provide a platform for people to communicate with and ease each other, especially those who are confined due to geographical or other limitations [43]. When people are linked in network relationship, the ability to maintain the urban resilience will notably increase even they confront complex problems [44].

Emergency response and daily management are considered to belong to rapidity. First of all, rapid emergency response following disasters is indispensable. This function will allow the provision of assistance for satisfying basic needs such as food and shelter [45]. In addition, some researchers argue that daily management is a relevant capacity to build resilience [45,46]. The management of the daily life determines the performance of a city in response to disasters, which places a substantial impact on urban rapidness [47]. Emergency response and daily management interact with each other. The ability of communities to collaborate with the society and to cooperate with various institutions, is a major focus of emergency response [48].

Medical resources and public service are considered to belong to redundancy. Adequate reserve supplies and well-organized emergency supply chain will guarantee a smooth operation of a city, thus enhance the redundancy of the community in the face of epidemic [49]. Representing a cornerstone to ensure the well-being of humankind, medical resources ensure the availability of health services [50]. Lack of resources is evidenced in the reaction towards the coronavirus spread around the world. Particularly, even the most advanced economies in the world also have trouble fulfilling the requirements of health services [51].

Experience in disaster and information technology are considered to belong to resourcefulness. Whether a community implements early warning to respond to disasters or innovative measures to prevent disasters, can be used to assess its disaster experience gained previously [52]. Regardless of previous disaster experience, information technology enables daily communication, in which case, it is convenient for people to gain reliable information regarding effective prevention and control measures timely.

### 2.3. Relationship of Influencing Factors

The ISM method was first proposed by Warfield to construct the hierarchical model of influencing factors in complex systems. By using this structural model, vague ideas, relations and views can be transformed into an intuitive model structure with good structural relations, which also delivers good and intuitive readability and reference [53].

Eight experts from higher education institutions in related fields are selected to weight and score the eight factors, of whom five are male and three are female, accounting for 62.5% and 37.5%, respectively. The interviewees include four professors, two associate professors and two lecturers. These experts conduct their own research in this field and their evaluations are fairly reliable. The matrix of relationships between the influencing factors is shown in Table 3.

**Figure 2:**
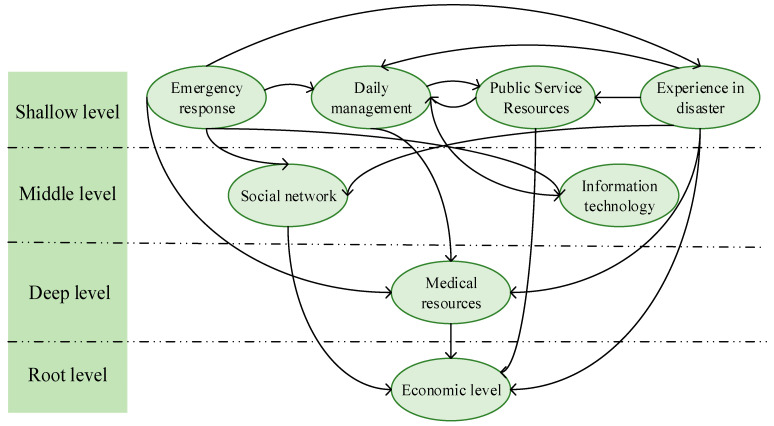
Hierarchical model.

Based on the results calculated with the MATLAB software, the ISM model is built, as shown in Figure 2.

### 2.4. Resilience Performance

The PROMETHEE method depicts the priority of a limited number of alternatives (individuals) on attributes through a set of evaluation criteria, and lists the advantages and disadvantages of each scheme according to the gap between the attribute values of each scheme. The set of influencing factors and the target communities are identified first, and then the performance of different communities under various influencing factors is assessed with the expert scoring method to obtain a weight matrix. Finally, a suitable preference function is selected in Visual PROMETHEE software. Integrated with the weights of influencing factors, the weight matrix is imported to analyze the results.

## 3. Case Study and Results

### 3.1. Evaluation of Community Epidemic Resilience

Taking Nanjing as an example, this paper selects four different communities under the jurisdiction of Meiyuan Street in Xuanwu District, including: Community F, S, T and D. The basic information of the communities is shown in Table 4.

Five staff of Meiyuan Street Office are invited to score the four communities with the Likert five-point scale method, on a scale from 1 to 5, as shown in Table 5.

**Figure 3:**
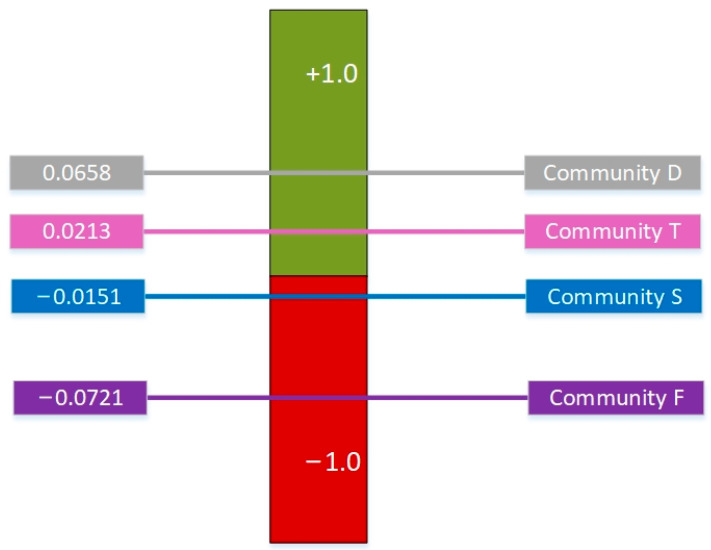
PROMETHEE II Complete Ranking.

The resilience of the four communities following COVID-19 is obtained as shown in Figure 3. The resilience of Community D performs the best; Community T follows closely; Community S ranks the third and Community F is ranked the last. The results are consistent with the actual disaster situation and perception.

As noticed from Table 5, social network has little influence on Community D and Community T. It is presumably contributed to its outstanding commercialization and relatively advanced economy. With the social elements enriched constantly, the social network has already reached a high level. In addition, Community D is prosperous and diverse, gathering many famous Nanjing attractions, such as the Presidential Palace, Nanjing Museum. Commercial buildings such as shops and malls, hotels and office buildings are also located here. In addition to residents, many businessmen and tourists frequent this community. 

TPM communities are predominated by administrative departments, with fewer residents, armed forces, forming relatively single and fixed social network relationships, so they are less affected by this. Most of this community is covered by the Nanjing Military Region, the affiliated family buildings, and the Nanjing Military Region General hospital. Soldiers are trained with high execution ability, strong unified command and coordination skills, and can quickly respond to emergencies. 

Community F is relatively geographically remote, occupied by old buildings with mostly elderly residents, who are closely related to each other. Consequently, it is significantly affected by the social network. Furthermore, since the elderly are relatively vulnerable, this community can be considered more sensitive to daily management and medical resources.

**Figure 4:**
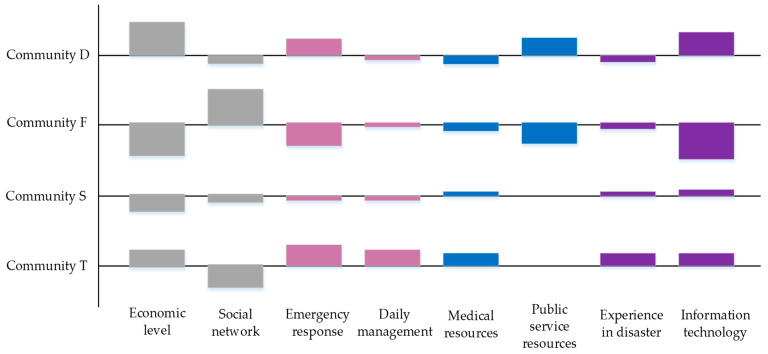
Strengths and weaknesses of each community.

Community S is less densely populated with several small residential areas. It accommodates residents with relatively high level of education, such as college students and retired teachers. The overall civilization is considerably higher than the other three communities. Considering its community structure, it has high requirements for information quality and transmission, so information technology poses the greatest impact, whereas economic level, social network and emergency response would exert negligible influences. The strengths and weaknesses of each community are shown in Figure 4.

The sensitivity analysis of influencing factors are as follows: (1) When the weight of economic level reaches 84% or public service resources reaches 94%, the desired resilience performance of Community F can be achieved. It demonstrates that the economic level and public service resources in Community F need to be developed urgently. (2) The social network is not a major influence to Communities F and S, however, increasing social network complexity in both communities without restriction will place a negative impact on community resilience once social network reaches 36%. (3) Community S is heavily influenced by emergency response and accordingly, a lack of prior emergency response will pose a major constraint on community resilience. (4) The information technology is not a major constraint on the epidemic resilience for Communities F and D. However, overemphasis on information technology does not increase the desired effect on community resilience. On the other hand, the Community T has a poor level of information technology, and only a fair increase in information technology will expectantly improve the resilience.

**Figure 5:**
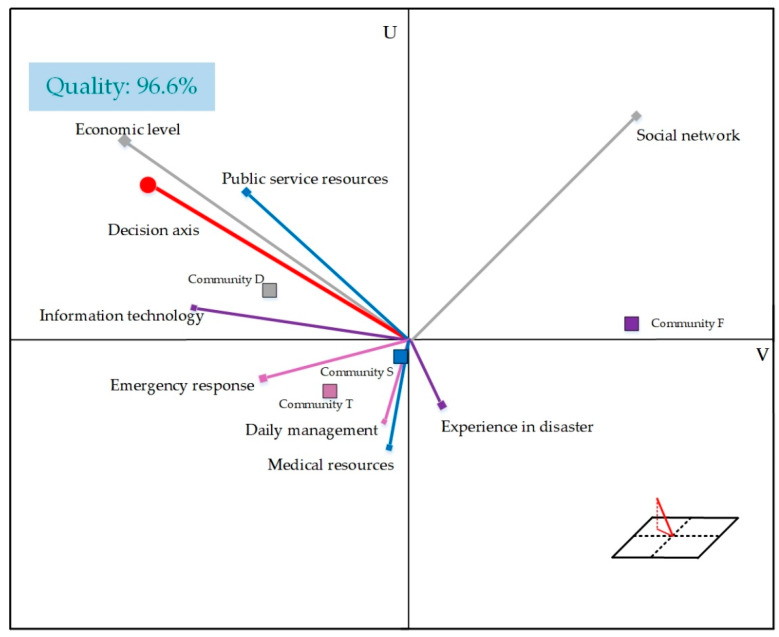
Performance of community epidemic resilience.

Graphical Analysis for Interactive Aid (GAIA) is a descriptive helper method for PROMETHEE as shown in Figure 5. The GAIA is a three-dimensional visual representation of the similarity between decision criteria and decision objectives in three planes, U-V, U-W and W-V. To allow information to be fully presented in the diagram, the U-V planes were chosen for illustration in this study. The “Quality: 96.6%” in the top left of the diagram indicates that the GAIA, when presented in the U-V plane, represents 96.6% of the information in 3D space. The thick red line at the endpoint denotes the decision axis. A longer line indicates that more information is expressed on the plane, and accordingly, the decision is more accurate. When the direction of the influence criterion is the same with or similar to that of the decision axis, it means that the influence criteria meet the current evaluation criteria. Specially, when economic level, public service resources and information technology are the main evaluation criteria, Community D performs the best. When the social network is the main evaluation criterion, Community F ranks the first. When emergency response, daily management and medical resources are the main evaluation criteria, Community T and S will be the targeted object.

### 3.2. Implements

- Robustness

It is imperative for the neighborhood committee of Community T to effectively improve prevention and control measures, and strengthen the governance and function of urban management department for better coordination and greater efficiency.

Considering that Community T is mostly a government department and military station, it is reasonably necessary to build a good relationship between the police and the people. Concurrently, these following measures will presumably enhance the community resilience, including, improve the cooperation between the sub-district office and the city management department, emphasize the mental health of residents, solve the conflicts among the residents in a timely and effective manner, and focus on specific groups of people.

The Community F performs poorly under economic indicators. After collecting 650 valid questionnaires in China, Zhang et al. also pointed out that communities with a higher level of economic development tend to be more resilient during the outbreak of the COVID-19 [1]. Therefore, it is crucial to improve the economic condition, such as expanding financial support to the community and attracting overseas investment to increase the epidemic resilience of the Community F. For the governments, certain funds against epidemic can be established to promote currency circulation in Community F under the epidemic to improve people’s livelihood.

- Rapidity

Linked to the ability to operate the system, rapidity is an essential element of the community resilience. Here, rapidity refers to emergency response and daily management. Community D performed well in emergency response, owing to its relatively solid economic foundation and rapid information transmission. The early warning department can use the neighborhood committee as the fundamental unit of disaster prevention and control, which can effectively improve the efficiency of information transmission. When data is questioned and inaccurate information is disseminated, community resilience will be largely challenged [54,55]. The emergency response is at the root level in the ISM analysis, but the emergency response performance of Community F is poor. Therefore, Community F needs to eliminate communication barriers in information transmission and improve the efficiency of the early warning department. 

Additionally, the community should necessarily strengthen the communication and connection with external volunteer groups and other social forces to enhance community resilience. All four communities are affected by daily management, which all perform satisfactorily. The reason for the investigation is that daily management is most closely related to people’s livelihood, and in the ISM analysis results, it is the primary source factor, which places a substantial impact on community resilience. All four communities highly value daily management, and the community governance is well-organized, which allows strong community resilience.

- Redundancy

In terms of medical resources, Community S and Community T exhibit excellent performance. Community S has a series of tertiary hospitals such as Zhongda Hospital, with advanced medical care and abundant medical resources, which have played a critical role in fighting the epidemic. Mainly based on military hospitals, Community T is highly unified, distributed with orderly and standardized medical resources. As the source indicator of community epidemic resilience, public service resources have an impact on all four communities, of which Community D performs the best, while T, Fand S communities perform the worst. Owing to its rapid development, numerous commercial streets, excellent economic conditions, and large flow of people, Community D has a large demand for, thus accordingly, a good reserve of public resources and excellent resilience in redundancy when dealing with major disasters. 

Community F contains numerous small old communities, where the facilities are aging and not updated timely. When dealing with sudden disasters, public service resources are not sufficient for it to respond well. From the study in Kerala India, Shine et al. similarly found that a rational redistribution of health care resources, endows Kerala with the highest recovery rate and lowest death rate among Indian states [16]. In this regard, government departments should pay more attention to the elderly group, conduct instant inspections, supplement the materials that community residents need during disasters, and cooperate with social caring service to expand the community’s epidemic prevention materials reserves.

- Resourcefulness

The disaster experience is at the root level, and all four communities which perform well are affected by it. It can be considered that the community has encountered disasters before and accumulated certain experience from them. Both the management and the residents of the community can learn from past disasters, which strengthens resilience for the community to fight against the epidemic. 

The community management department can regularly hold disaster-related knowledge sharing activities such as disaster drills, publicity and education, and general science lectures in the community, to improve residents’ skills against disasters. In terms of information technology indicators, Community F has the worst performance. Considering the aging of the community, the acceptance of new information is slow. Neighborhood committees can set up information display board in the community to update the latest news.

Generally, Community D has performed best, with effective information communication and sufficient material reserves. Community T performed relatively well among the four communities, with numerous small groups, extensive contacts with the community police, and strong action skills. But the problem is that the power and authority within the community are relatively centralized, which leads to inefficient transmission of information, thus attention should be paid to strengthening association with external organizations. Regarding economic indicators, Community F lacks financial support, and the connection between various stakeholders in community liaison is sparse and not close, resulting in obstruction of information transmission.

## 4. Conclusions

The improvement of community resilience is a major research topic in the context of COVID-19. By researching existing studies, it is found that there are many factors that can possibly influence community resilience. This paper finds through ISM analysis that community resilience is mainly influenced by eight factors, of which emergency response, daily management, public service resources and experience in disaster are the root factors. Finally, this study evaluates the performance of epidemic resilience using PROMETHEE II method through empirical cases in Nanjing, China.

Three main implications were identified from this study, which are critical for stakeholders and decision-makers in China to incorporate into their resilience planning:

(1) Rational allocation of public resources

The level of health care is one of the most important influences in determining the resilience of a community during a new crown epidemic. Communities with more resourcefulness are able to provide sufficient material resources to cope with the epidemic; contrastingly, is it pressing to provide sufficient medical resources for those communities with poor location and aging residents. Therefore, it is necessary to redistribute health care resources for different communities. Ensuring the adequacy of public resources in the community is an effective initiative to improve community resilience.

(2) Ensure the accuracy of information delivery

Noted from the case study, the resilience of Community can be strengthened by improving the stable transmission of effective information and interdepartmental synergies while preventing the spread of the virus during the COVID-19. This shows that ensuring the accuracy as well as the timeliness of information will greatly booster community resilience.

(3) Conduct disaster learning

From the case above, it is found that emergency response placed a significant impact on community resilience. Lack of prior emergency response can exert a major constraint on the level of community resilience. Significantly improving emergency response can effectively increase the level of community resilience. Communities need to ensure that lessons are learned from previous disasters and that associated changes are made immediately in the aftermath. Only then will it be possible to effectively implement changes in practice for post-disaster management and thus improve pandemic preparedness.

Overall, community resilience is multi-dimensional and multi-factorial, and thus it requires an improved level of integration to essentially strengthen community resilience. The research framework and the several implications proposed in this paper provide a practical reference for other countries around the world to apply to improve community resilience during COVID-19. Nonetheless, there is still much relevant work to be done in the future. First, a limited number of influencing factors have been fully examined in this study, and other factors might be considered in the future to reveal additional insights. Second, the proposed improvement paths for community resilience can be evaluated through simulation techniques in future studies to test the validity.

## Figures and Tables

**Table 1:** Principal component and cumulative contribution rate.

Item	Eigenvectors							
Q1	0.136	0.395	−0.363	0.137	0.132	0.205	−0.002	−0.284
Q2	0.172	0.484	−0.108	0.005	−0.006	0.011	−0.054	0.013
Q3	0.182	0.385	−0.302	0.194	0.107	−0.116	−0.170	−0.071
Q4	0.175	0.057	0.148	0.247	−0.406	0.420	0.097	−0.191
Q5	0.238	0.045	0.216	0.040	0.251	−0.191	−0.017	0.273
Q6	0.216	−0.015	0.340	0.162	−0.078	0.102	0.081	−0.281
Q7	0.259	0.116	0.203	−0.416	0.081	0.126	0.008	0.148
Q8	0.087	0.173	0.392	0.431	0.247	−0.076	−0.143	0.240
Q9	0.306	−0.043	0.018	0.212	0.000	0.042	−0.040	0.145
Q10	0.260	0.029	0.047	0.103	−0.311	0.011	0.322	0.226
Q11	0.138	0.296	−0.070	−0.266	−0.428	−0.135	0.244	0.382
Q12	−0.021	0.294	0.271	−0.347	−0.187	−0.313	−0.414	−0.280
Q13	0.154	−0.032	0.111	−0.360	0.145	0.648	−0.169	0.024
Q14	0.131	−0.271	−0.298	0.108	−0.419	−0.116	−0.218	0.050
Q15	0.171	−0.150	0.158	0.166	−0.240	0.023	−0.586	0.081
Q16	0.257	−0.155	−0.262	−0.191	0.003	−0.070	−0.296	0.025
Q17	0.276	−0.125	−0.251	0.035	0.233	0.072	−0.014	0.160
Q18	0.272	−0.039	0.185	−0.031	0.053	−0.321	0.225	−0.288
Q19	0.298	−0.106	0.022	−0.198	0.070	−0.122	0.148	−0.135
Q20	0.279	−0.226	−0.109	−0.049	0.195	−0.099	0.011	0.096
Q21	0.279	−0.188	−0.043	−0.002	0.016	−0.100	0.120	−0.448
Eigen value	8.072	2.436	1.702	1.341	1.250	1.202	0.977	0.821
Contribution rate	0.384	0.116	0.081	0.064	0.060	0.057	0.047	0.039
cumulative contribution rate	0.384	0.500	0.581	0.645	0.705	0.762	0.809	0.848

**Table 2:** Determination of influencing factors of community epidemic resilience.

Influencing Factor		Principal Component							
		1	2	3	4	5	6	7	8
C1	Economic level								√
C2	Social network						√		
C3	Emergency response			√					
C4	Daily management	√							
C5	Medical resources							√	
C6	Public service resources		√						
C7	Experience in disaster					√			
C8	Information technology				√				

**Table 3:** Relation matrix of influencing factors.

		Robustness		Rapidity		Redundancy		Resourcefulness	
		C1	C2	C3	C4	C5	C6	C7	C8
**Robustness**	C1	0	1	0	0	1	1	0	1
	C2	0	0	1	0	0	0	1	0
**Rapidity**	C3	0	0	0	1	0	0	0	0
	C4	0	0	0	0	0	1	1	0
**Redundancy**	C5	0	0	1	1	0	0	0	1
	C6	0	0	1	1	0	0	1	0
**Resourcefulness**	C7	0	0	1	0	0	0	0	0
	C8	0	0	1	1	0	0	0	0

**Table 4:** Basic information of the communities.

Community	Permanent Residents	Area (km^2^)	Features
D	11,000	0.45	commercial areas, office buildings, memorials, district government, houses
S	8000	0.5	students, teachers and other senior intellectuals
T	20,000	1.4	military compound contained

**Table 5:** Epidemic resilience adapting scoring scale.

Influencing Factors		Community			
		D	S	T	F
Robustness	Economic level	5	3	4	2
	Social network	3	3	2	5
Rapidity	Emergency response	4	3	4	2
	Daily management	4	4	5	4
Redundancy	Medical resources	4	5	5	4
	Public service resources	5	4	4	3
Resourcefulness	Disaster in experience	4	4	5	5
	Information technology	5	4	4	2

## Data Availability

The data presented in this study are available on request from the corresponding author. The data are not publicly available due to privacy.

## Appendix A

**Table A1:** Primary Selection of Indicators and Frequency [1,3,7,12,13,21,22,23,24,25,26,27,28,29,43,52,56,57,58,59,60,61,62,63,64,65,66,67,68,69,70,71,72,73,74,75,76,77,78,79,80].

Dimension	Frequency	Included Indicators	Frequency
Resource	22	Public health resource	7
		Resource for essential need	6
		Material resource	5
		Emotional resource	2
		Protective equipment	2
Facility	18	Infrastructure	7
		Emergency facility	4
		Regional industrial structure	3
		Diagnostic facility	3
		Isolation ward	1
Perception	15	Perception of personal risk	5
		Knowledge in preparation for events	5
		Perception of surviving	2
		Awareness of the economic crisis	2
		Respect to social distance	1
Health	14	Mental health	5
		Health care	4
		Health service	3
		Healthcare disparity	2
Internet	13	Internet access	6
		Safety net	3
		Social network	2
		Resilient information network	2
Financial	12	Financial aid	4
		Economic crisis	3
		Personal cost	3
		Economic security	2
Support	10	Social support	3
		Emotional support	3
		Prosocial act	2
		Guideline for protection	1
		Psychological attribute	1
Resident	9	Personal connection	3
		Individual quarantine	2
		Neighborhood association	2
		Older adult’s anxiety	2
Equality	9	Resource equality	3
		Racial equality	2
		Educational equality	2
		Income equality	2
Medical treatment	9	Mental health service	4
		Number of medical personnel	3
		Mortality in nursing home	2
Social distance	8	Vulnerability of population	2
		Living condition	1
		The use of mask	1
		Geographic location	1
Workforce	7	Unemployment rate	3
		Workforce absenteeism	2
		Stable workforce	1
		Work performance	1
Public	7	Public health messaging	4
		Public health system	2
		Public virtual information	1
Capacity	7	Capacity for transformation	2
		Capacity for health emergency preparedness	2
		Capacity for health emergency response	1
		Capacity for innovation	1
		Capacity for agility	1
Knowledge	6	Strategic planning knowledge	3
		Disaster learning	3
Massage	6	Mismanaged fatality	2
		Language barrier	2
		Misinformation addressing	1
		Free flow of information	1
Trauma	5	Transmissibility of the disease	3
		Psychological trauma	1
		Compassion fatigue	1
Government	5	Government support scheme	3
		Government guidance	2
Education	4	Educational disruption	3
Culture	4	Cultural practice	2
		Religious practice	1
Collaboration	3	Cooperatives’ embeddedness	1
Productivity	3	Productivity among official and employee	1
Compliance	3	Individual compliance	2
		Community compliance	1
Organization	3	Warning mechanism	1
		Decision-making skillset	1

## Appendix B. Questionnaire on Key Factors of Community Resilience

Dear interviewee, the data and information collected in this questionnaire will only be used for academic research, and personal information will not be disclosed.

Thank you for your assistance!

Community Resilience Governance Research Team, Engineering Management Department, Nanjing Forestry University

**The first part**—The basic information

1. Your gender: [Single topic selection]

○Male ○Female

2. Your age: [Single topic selection]

○Under the age of 18 ○18~3 ○36~60 ○More than 60 years of age

3. Your education level is: [Single topic selection]

○High school and below ○Specialized subject

○Undergraduate course ○Postgraduate and above

4. Is there anyone in your community who has ever been infected with novel Coronavirus? [Single topic selection]

○Yes ○No

5. What role do you play in the community? [Single topic selection]

○Member of the community residents committee

○Community residents

○Property service center staff

○Community security

○Community police

○Government staff

○Street office staff

○other

**The second part**—Factors influencing urban community response to COVID-19

6. During the COVID-19 outbreak, medical, sanitation and service facilities in and around the community have operated normally. [Single topic selection]

○Completely incompatible ○Not quite ○generally

○Basically meet ○Completely suitable

7. During the COVID-19 pandemic, there is no shortage of daily necessities in your community, such as food, oil, masks, and fresh fruits and vegetables. [Single topic selection]

○Completely incompatible ○Not quite ○generally

○Basically meet ○Completely suitable

8. During the COVID-19 outbreak, fitness, leisure, convenience and service facilities in the community function well and are in normal use. [Single topic selection]

○Completely incompatible ○Not quite ○generally

○Basically meet ○Completely suitable

9. During the COVID-19 outbreak, the communication system within the community was smooth and stable, and communication with the outside world was barrier-free. [Single topic selection]

○Completely incompatible ○Not quite ○generally

○Basically meet ○Completely suitable

10. Before and after the outbreak of COVID-19, community residents have never been lax in their protection and have a strong sense of personal protection. [Single topic selection]

○Completely incompatible ○Not quite ○generally

○Basically meet ○Completely suitable

11. After the outbreak of COVID-19, the community immediately took measures to carry out personnel inspection, isolation and treatment, and increase publicity. [Single topic selection]

○Completely incompatible ○Not quite ○generally

○Basically meet ○Completely suitable

12. During the COVID-19 pandemic, volunteers, for-profit/non-profit organizations and other social forces provided timely and effective assistance and support to communities. [Single topic selection]

○Completely incompatible ○Not quite ○generally

○Basically meet ○Completely suitable

13. Individuals and families have remained physically and mentally healthy and unaffected by COVID-19. [Single topic selection]

○Completely incompatible ○Not quite ○generally

○Basically meet ○Completely suitable

14. During the COVID-19 pandemic, communities have provided convenience services to help solve problems such as delivery, movement of people and the use of public facilities. [Single topic selection]

○Completely incompatible ○Not quite ○generally

○Basically meet ○Completely suitable

15. During the COVID-19 outbreak, the price level around the community was relatively stable without major fluctuations. [Single topic selection]

○Completely incompatible ○Not quite ○generally

○Basically meet ○Completely suitable

16. Individual and household incomes have not been significantly affected before and after COVID-19. [Single topic selection]

○Completely incompatible ○Not quite ○generally

○Basically meet ○Completely suitable

17. There was no significant difference in individual and household spending during the COVID-19 pandemic. [Single topic selection]

○Completely incompatible ○Not quite ○generally

○Basically meet ○Completely suitable

18. During the COVID-19 outbreak, community residents and disaster management personnel in the community have maintained a harmonious relationship, close contact, communication and harmony. [Single topic selection]

○Completely incompatible ○Not quite ○generally

○Basically meet ○Completely suitable

19. During the COVID-19 outbreak, the traffic around the community remained good and travel was not affected. [Single topic selection]

○Completely incompatible ○Not quite ○generally

○Basically meet ○Completely suitable

20. During the COVID-19 outbreak, community residents can actively respond to various epidemic prevention policies issued by the government and obey the unified management of the community. [Single topic selection]

○Completely incompatible ○Not quite ○generally

○Basically meet ○Completely suitable

21. During the COVID-19 pandemic, the number of unemployed in the community has not significantly increased, and the employment rate is stable. [Single topic selection]

○Completely incompatible ○Not quite ○generally

○Basically meet ○Completely suitable

22. During the COVID-19 outbreak, community residents have already had good basic attempts on the transmission mechanism of the virus and epidemic prevention measures. [Single topic selection]

○Completely incompatible ○Not quite ○generally

○Basically meet ○Completely suitable

23. Prior to COVID-19, communities already had relevant experience or contingency plans to deal with major infectious disease events, which can be applied to the current outbreak. [Single topic selection]

○Completely incompatible ○Not quite ○generally

○Basically meet ○Completely suitable

24. During the COVID-19 outbreak, communities are equipped with digital information means to timely release epidemic information and inform community residents of epidemic trends and instructions. [Single topic selection]

○Completely incompatible ○Not quite ○generally

○Basically meet ○Completely suitable

25. During the COVID-19 pandemic, communities received financial or material subsidies from the government. [Single topic selection]

○Completely incompatible ○Not quite ○generally

○Basically meet ○Completely suitable

26. Community health care workers and security personnel are fully staffed during the COVID-19 pandemic. [Single topic selection]

○Completely incompatible ○Not quite ○generally

○Basically meet ○Completely suitable
